# Long-Term Trends in Changes in Physical and Motor Development Observed among Physiotherapy Students from Bydgoszcz in 2011–2020

**DOI:** 10.3390/ijerph192114444

**Published:** 2022-11-04

**Authors:** Andrzej Lewandowski, Zuzanna Piekorz, Jadwiga Sarwińska, Marcin Siedlaczek

**Affiliations:** Department of Physiotherapy, Faculty of Health Sciences, Ludwik Rydygier Collegium Medicum in Bydgoszcz, Nicolaus Copernicus University in Toruń, 87-100 Toruń, Poland

**Keywords:** male and female students, morphological characteristics, BMI and WHR index, Cooper test, 2 km march, spinning coordination, dimorphic differences, environmental dependencies

## Abstract

Introduction: The aim of the study is to assess the changes in somatic and motor characteristics in adolescents studying physiotherapy from 2011–2020. It was hypothesized that there was no secular trend of morphological features, its maintenance in endurance, increased sexual dimorphism and the influence of the COVID-19 pandemic on the observed characteristics. Materials and methods: Young people studying at the Collegium Medicum in Bydgoszcz were examined. Basic somatic features, endurance and coordination were measured. WHR, BMI and dimorphism indices were calculated based on the results from 538 female and 217 male examined students. Results: There was a decrease in body height with an increase in body weight in relation to the values recorded in the years 2001–2010 (BMI: women D = 0.49, Z = 2.9192; men D = 0.93, Z = 3.5746; WHR women D = 0.01, Z = 2.88491; men D = 0.02, Z = 3.5746), an increase in sexual dimorphism and a significant increase in the waist circumference of women (R2 = 0.008, *p* (F) = 0.0353) year by year by 0.3099 cm (*p* = 0.035). Conclusions: The obtained results and the comparisons made allow us to assume that some people studying physiotherapy may have problems with meeting the standards that require physical effort.

## 1. Introduction

The profession and work of a physiotherapist, as well as the education process, are undergoing multidirectional changes. They are caused both by the development of medical knowledge and various social expectations [1,2,3,4,5,6]. Despite the fact that they are closer to the medical profession, physical effort and its negative effects are an inherent component of a physiotherapist’s work [7,8,9,10]. These changes also mean that the higher education of the previously feminized profession is more often chosen by men, and the basic morphological features of adolescents studying physiotherapy and their sexual dimorphism, even in the light of long-term observations, do not change much [11,12]. It is also known from our own research that from the social perception, a high level of physical fitness is expected from a physiotherapist. For the successful performance of many therapeutic standards, in addition to a wealth of directional knowledge and many motor skills, endurance and motor coordination are the most important indications of motor skills [10,13]. They also allow a physiotherapist to effectively oppose the numerous health threats occurring among people working in this profession, which most often relate to degenerative diseases of individual spine sections of the spine, as well as of the upper limbs [7,8,9,14]. Our previous long-term research of adolescents studying physical therapy indicate a tendency to improve running endurance measured with the Cooper test with a decrease in the level of rotational coordination, especially among female adolescents [12]. Thus, it can be assumed that more and more men studying physiotherapy are slightly better prepared than their female colleagues to meet professional standards that require a lot of physical effort. Extensive literature data on trends in secular somatic traits and signs of motor skills indicate their continued occurrence in many population groups, despite the progressing process of equalizing living conditions [15,16,17,18]. Considering the above, research on the changes in somatic features and physical fitness of future physiotherapists is advisable. They can be helpful in updating the program of physical education and the method of enrollment in physiotherapy studies. Thus, the aim of this study was to attempt to determine the size and directions of changes in the basic somatic and motor characteristics of females and males studying physiotherapy in the past decade, as well as an attempt to relate them to social and environmental conditions. Taking into account the literature data, as well as the results of our earlier research from the first decade of the 21st century, cited above, it was assumed that there was no clear occurrence of a secular trend in the values of morphological features and its maintenance in terms of running endurance, as well as the further deterioration of motor coordination. It was also hypothesized that the beginning of the COVID-19 pandemic and the related lockdowns (20 March–2 April) and the complete closure of universities (11 March–24 March), as well as remote teaching until the end of the academic year, resulted in a significant increase in body weight, and thus in the size of BMI indicators and WHR, as well as a significant decrease in the level of endurance which proves the deterioration of some determinants of health even among relatively fit and healthy adolescents, whom physiotherapy students can be considered as [19].

## 2. Materials and Methods

### 2.1. Study Design

The research covered young females and males studying physiotherapy at Collegium Medicum in Bydgoszcz, UMK in Toruń from 2011–2020. The data were collected during classes in movement education and teaching movement methodology, conducted initially in the first and, from 2015, in the second year of undergraduate studies. For this reason, in 2014, the initiated observation was not carried out, which from 2019 already covered the participants of master’s studies. Thus, the students of subsequent years were tested once. Each year, new observation participants were assessed. All groups of adolescents included in the study did not have a fitness exam at the time of university enrollment and, with the exception of the last group, they followed a similar program of compulsory physical education and movement education with the methodology of teaching movement. The lack of the fitness examination, which was used in the previous decade, could be a significant reason for the expected morphological and motor changes in students of the currently observed years

### 2.2. Study Procedure and Instruments

At the end of each academic year, in addition to waist and hip circumference, height and weight were measured as basic morphological features. BMI and WHR were calculated separately for each participant [20]. This allowed for the assessment of the secular trend of these features in the group of physiotherapy students under study. It is still present in the general population of young people, in contrast to some selected groups. Gross motor endurance was measured by the Cooper test and a two-kilometer walk; its levels are strongly correlated with VO2max. The Staroste test was used to assess the right and left rotation coordination [21,22,23].

The calculated Mollison indexes were used to assess the sexual dimorphism of the assessed traits in the subsequent years of observation [24]. Except for the research of the last group, the students of which carried out feasible measurements and performance tests themselves, all measurements were made by a specialized research team under the same conditions and in accordance with the test procedures. In total, the results were obtained from 538 female students and 217 male students, which corresponded to 96% of the study’s population. The characteristics covered in this study were analyzed, including the basic sociometric features, by comparing them with those which characterized the adolescents who studied physiotherapy in the previous decade [12].

### 2.3. Data Analysis

The chi-square test of independence was used to check whether the percentage of women and men in the study’s population changes with time. Student’s *t*-test for independent attempts was used to assess the differences of the average results of women and men in particular years of the study. To compare the size of the groups and the average results of observations from both decades, the test of the difference between uncorrelated proportions, independent attempts of the Z-test, which is an asymptotic counterpart of Student’s *t*-test (for groups with more than 100 observations), were used. Changes in results over time were assessed using the linear regression analysis and nonparametric equivalents of the variance analysis—the Kruskal–Wallis H test and the Mann–Whitney U test with the Bonferroni correction (for post hoc comparisons) due to the failure to meet the assumptions of the classic ANOVA. The analysis was performed in Python 3.8.10 (drawing on work Fluent Python of authorship Luciano Ramalcho, published by O’Reilly Media, Inc., Sebastopol, CA, USA in 2015), setting the significance level alpha = 0.05.

## 3. Results

**Figure 1:**
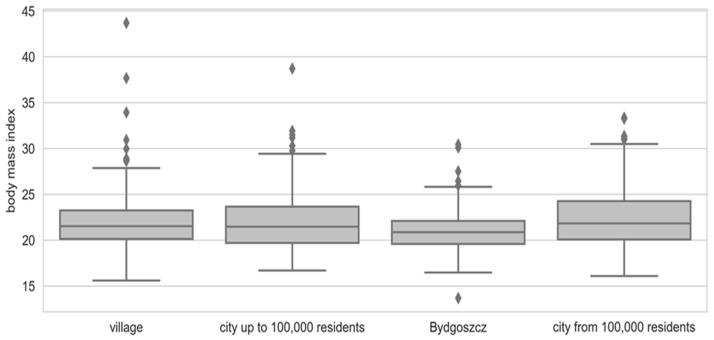
Graphical characteristics of the BMI of the surveyed women differing in the size of the place of residence. The chart shows: minimum, first quartile, median, third quartile, maximum, extreme observations and atypical observations. ⬪—extreme observations and atypical observations.

The characteristics of descriptive statistics imply that almost all of the researched women and men graduated from general secondary schools (95.86%) and, in similar participation, lived in rural areas (36.44%), cities with up to 100,000 inhabitants (33.68%) and larger cities (29.88%), half of whom were residents of Bydgoszcz (14.25%). The percentages of their parents’ education levels were different among mothers and fathers. Mothers most often had higher education (43.10%) and fathers—vocational education (39.88%). The percentages of mothers and fathers with secondary education were similar (36.55% and 32.87%). The lowest percentage of mothers had vocational education (20.34%), and so was the number of fathers with higher education (27.24%). There was no significant influence of demographic factors on the analyzed characteristics, except for a lower BMI (21.17 ± 2.94) of Bydgoszcz women compared to the young women from other large Polish cities (*p* = 0.0389). Figure 1 present characteristics of the BMI of the surveyed women differing in the size of the place of residence.

The demographic characteristics of the observation’s participants are presented in Table 1.

As shown in the Table 1, no statistically significant relationship was found between the year of the study and gender (chi2 = 6.698, *p* = 0.569) and the age change of the studying youth (F = 0.767, *p* = 0.381). However, a statistically significant difference in the age of women and men older than those women was found (t = −2.17, *p* = 0.03). The comparison of the participations of women and men and studying groups in the decade from 2011–2020 showed no differences, and in the case of the lower average age of the currently observed youth, the differences were statistically significant.

The anthropometric characteristics of the studied adolescents are presented in Table 2 and Table 3.

With the exception of the average hip circumferences of three different student groups, the remaining ones indicate a statistically significant difference between the average values of men and women.

The comparison of the average measurements and indexes with those of the youth studying in the previous decade, except for the body height in the groups with both genders and the women’s hip circumference, showed higher values of all the characteristics of the currently observed group of students.

Table 4 presents the results of the performed fitness tests and the assessment of the average differences from both decades.

The average Cooper test results for all groups of students were higher than for female students, and the 2 km walk times were lower, which also means a better result. No statistically significant difference was found in the marching attempt of the student groups surveyed in 2016 or in almost all of the average results of the rotation coordination, as well as in the comparison of the averages for 2011–2020 with the results from the previous decade.

The results of the material developed in this way as well as the main purpose of the work required further elaboration of the material with the use of linear regression analysis. The somatic features are listed in Table 5, showing the statistical significance of the model in bold.

Table 5 shows, for the dependence of body weight on the age of students, the linear regression model is statistically significant only in the group of men (*p* (F) = 0.0147), with the determination coefficient of 0.024, which means that it does not explain its variability to a large extent. Each year, men were heavier by an average of 0.7498 kg (*p* = 0.015). In the group of women, there was no variation in body weight over time, which was similar to the height of both sexes. Thus, the regression model explaining the variability of BMI over the years clarifies it to a small extent only in the group of men (*p* (F) = 0.0415, R2 = 0.017), the value of which increases by an average of 0.1757 (*p* = 0.041) each year.

Linear regression models explain the variability of the waist circumference over the years in the group of women (R2 = 0.049, *p* (F) < 0.001) and men (R2 = 0.041, *p* (F) = 0.001) to a small extent. In the group of women, it increases annually by 0.7348 cm (*p* < 0.001), and in the group of men by 0.7015 cm (*p* = 0.001).

In the groups of both genders, the linear regression model shows the relationship between the hip circumference and the passage of time. The model for women (R2 = 0.010, *p* (F) = 0.014) shows that each year women have a larger hip circumference by 0.3054 cm (*p* = 0.014), while men are by 0.3158 cm (*p* = 0.045). Thus, the models are important for the value of the WHR index, but they poorly explain its variability for both women (R2 = 0.046, *p* (F) < 0.001) and men (R2 = 0.035, *p* (F) = 0.003). Each year its value increases for women by 0.0056 (*p* < 0.001) and for men by 0.0044 (*p* = 0.003).

In order to check whether the results from the individual years of the research differ significantly from the others, an analysis of variance (ANOVA) was performed separately for men and women.

The assumption of the analysis of variance with the groups being equal in number was not met (chi2 = 16.846, *p* = 0.032). Therefore, the Kruskal–Wallis H test was performed, the result of which (H = 53.266, *p* = 0.0) indicates that there are years which differ significantly from each other.

**Figure 2:**
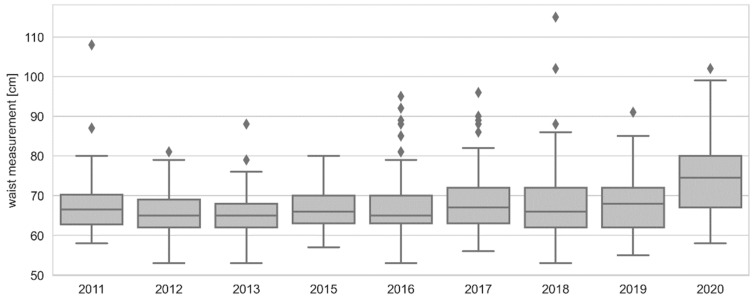
Graphical characteristics of temporal changes in women’s waist circumference. The chart shows: minimum, first quartile, median, third quartile, maximum, ⬪—extreme observations and atypical observations.

Multiple comparisons with the non-parametric Mann–Whitney H test with the Bonferroni correction prove that in the group of women, the waist circumference median in 2020 was the highest and differed significantly from the medians from the remaining years. The comparisons of the results from the remaining years did not show any significant differences, as shown in Figure 2.

**Figure 3:**
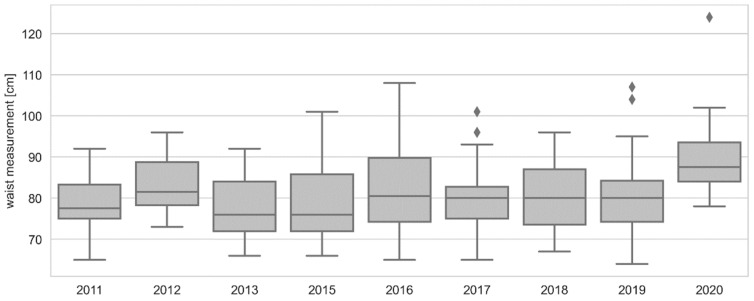
Graphical characteristics of temporal changes in men’s waist circumference. The chart shows: minimum, first quartile, median, third quartile, maximum, ⬪—extreme observations and atypical observations.

In the male groups, the assumption of the analysis of variance based on the groups equality in number was met (chi2 = 1.976, *p* = 0.982), as well as the assumption of the equality of variance (Levene’s test, st = 1.5, *p* = 0.158). However, the assumption about the normality of the distribution in the groups was not met because in 2019 and 2020, the distributions significantly differ from the normal distribution (Shapiro–Wilk test, *p*-values < 0.05). A non-parametric analysis of variance (H = 29.078, *p* < 0.001) proves that there are years with significantly different results. Multiple comparisons showed that the median waist circumference in 2020 was the highest and, with the exception of three years of research, differed significantly from the median measurements from the remaining years, as shown in Figure 3.

Due to the higher values of the waist circumference of men and women in 2020 compared to the others, it was omitted in order to check whether the linear dependence of this feature on the year of the study will be maintained. The results are shown in Table 6.

As can be seen from the table, the linear model for women is still significant (R2 = 0.008, *p* (F) = 0.0353), however, it has a lower slope value. On average, women’s waist circumference increased by 0.3099 cm annually (*p* = 0.035). For men, the model is irrelevant (R2 = 0.001, *p* (F) = 0.574.

The results of the regression analysis for the results of the fitness tests are presented in Table 7.

As can be seen from the table, the linear regression model for women slightly explains the variability of the Cooper test value (R2 = 0.021, *p* (F) = 0.000316), which decreases each year by −16.7312 m (*p* < 0.001). In the group of men, the linear regression model was insignificant, as in the case of the results of the 2 km walk for women (R2 = 0.002, *p* (F) = 0.280) and men (R2 = 0.003, F = 0.5729, *p* (F) = 0.450)

The variability of the right rotation coordination measurement was slightly dependent on time in the group of women (R2 = 0.008, *p* (F) = 0.0287). Every year, its value decreased on average by −2.0632 (*p* = 0.029), and in the group of men, no similar relationship was found (R2 = 0.013, *p* (F) = 0.0974). The left-hand coordination variability was also slightly explained in the group of women (R2 = 0.008, *p* (F) = 0.0380) and men (R2 = 0.044, *p* (F) = 0.00173). For women, its value decreased by −1.8340 (*p* < 0.038) year by year, and for men by −5.2757 (*p* < 0.002).

The value of the Mollison index of the studied traits of female and male students, which allows the assessment of dimorphic differences in the subsequent years of the study, is presented in Table 8.

Mollison index (magnitude of dimorphic differences of the examined features)

*WD* = *M* ♀ − *M*♂ *S*♂

*M* ♀—arithmetic mean of the characteristics of the examined group of women

*M* ♂—arithmetic mean of the characteristics of the studied group of men

*S* ♂—standard deviation of the characteristics of the examined group of men

Dimorphism indexes of studied traits in subsequent years of observation have different values, and thus, they reflect the changes directions ambiguously. Minor increases were identified in the dimorphic differences of body height, the 2 km walk results and the rotation coordination, to the right side, in particular. The averages of the dimorphism index of the studied traits, except for the 2 km walk results and left-side rotation coordination, were slightly higher than those observed with the physiotherapy students from the previous decade and were more visible in the level of endurance measured with the Cooper test.

## 4. Discussion

The obtained research results allow us to make basic observations; however due to their relative value, we are required to refer to the results of observations from the previous decade as well as to the wider literature data.

The analysis of the basic anthropometric determinants of the sex and age structure of people studying physiotherapy and relating them to the situation from the previous decade shows that they do not change significantly, with a higher average age of male adolescents [12]. The clearly smaller and constant participation of male youth compared to the female students in physiotherapy studies, which we also observed in the previous decade, indicates the continuation of the process of feminization of medical professions in Poland, which may be related to the low economic attractiveness of working in these professions. Among physiotherapists, this situation may in the future lead to the process of marginalizing or avoiding therapeutic standards requiring physical effort, to which men are more predisposed than women and, as currently examined, characterized by significantly lower averages of anthropometric characteristics than for men covered by the observation [25]. However, with the exception of the body height of both sexes and the female hip circumference, they are larger than those found in the previous decade. Thus, it may indicate the process of changes in the body shape of future physiotherapists to a less expected and less favorable one in relation to the one predisposing them during physical exercise, which is indicated by people working in this profession [26]. The surveyed men are also characterized by the more favorable results of endurance tests, which similarly to the women’s and the average rotation coordination tests, do not differ from those measured in the previous decade. This observation, in light of the observed increase in body weight and circumference of the currently examined adolescents, seems to be a concerning phenomenon, which may indicate a decrease in the fitness abilities of future physiotherapists. The above assumption may also apply to the young females and males from other Polish academic institutions educating in physiotherapy, which do not commonly use the fitness examination to study this field. Such assumptions indirectly justify the lack of influence of basic environmental factors, claimed in the current research, such as the size of the inhabited environment, parents’ education and the type of secondary school graduation on the size of the studied characteristics. In this respect, the environment of physiotherapy students seems to be homogeneous, which we found earlier among people studying medicine and, through self-identification for the profession, more strictly selected from groups coming from lower social levels [27]. This thesis, however, requires verification because it is in contradiction with the significantly lower BMI of women from Bydgoszcz compared to the examined students living in other large Polish cities. Thus, with the highest body height and the lowest body weight, the examined women from Bydgoszcz distinguished the predictor of life expectancy and its quality to the greatest extent [28].

The basic observations are confirmed by the assessment of the temporal changes in most of the studied characteristics, which also allows us to verify the hypotheses. The lack of dependence of changes in body height in the studied adolescents over time indicates the disappearance of the secular trend of this feature, which still occurs in the general population of adolescents [15,16,29,30]. Therefore, in the light of the increase in body weight and hip circumference of men over time, as well as the size of the BMI and WHR index, as well as the increase in both circumferences among women, which also influenced the temporal changes in WHR, we consider the observed relationships to be unfavorable. We justify this statement with the results of studies showing the relationship between the length and quality of life and greater body height and lower weight, and thus BMI [28]. Considering the above, it can be concluded that the hypothesis concerning the extinction of trends in secular somatic features was confirmed only in terms of body height. However, it should be noted that the models of linear regression analysis, although significant, explained the variability of these characteristics to a small extent. The observed male response to lockdowns is also noteworthy, manifested by a significant increase in waist circumference that was no longer dependent on time after removing the results of the 2020 study. Among women, the dependence of this feature on time was significant. Thus, in the currently presented studies we have confirmed the phenomenon of males’ greater reactivity to environmental stimuli [31,32].

The observed changes in somatic features allowed us to expect similar dependences on time in the tested motor characteristics. They were more prominent in women’s groups, where the Cooper’s test averages decrease over time, as does both side-to-side and left-hand rotation coordination in men’s groups. These changes, and their lack in the previous decade, are explained by the resignation of the fitness exam for physiotherapy studies. Moreover, the tendency for the Cooper test result to deteriorate over time among women and the unfavorable changes in basic somatic features becomes a concerning phenomenon, especially when they constitute the vast majority in the studies and profession of a physiotherapist. This view is not changed by the lack of time changes in the results of the 2 km walk, which we also found in the previous decade, and we explain the reasons for the lack of correspondence in the endurance tests results by the specificity of the walking effort and its widespread use, and thus, to a small extent identified by participants as an exercise of endurance character [12]. Hence, the hypothesis put forward regarding the unfavorable temporal changes in motor characteristics has been positively verified only in terms of the results of the research on women. In the case of temporal changes in motor characteristics, linear regression models are also important, yet similarly to the case of somatic features, they also explain their variability to a small extent.

A necessary explanation should be provided for the Cooper test result of young people of both sexes examined in 2020, i.e., in the first half of the COVID-19 pandemic year, there was a month-long hard lockdown and a three-month remote studies continuation. According to the adopted hypothesis, we expected a decrease in its value, which turned out to be clearly more favorable among both women and men than in the vast majority of previous years of observation. We believe that the reason for such a study result could be due to measurement errors resulting from the independent attempt implementation by the examined youth. However, it is possible that the pandemic situation and the relatively short lockdown time significantly influenced the young people’s willingness to take up physical activity and increased the motivation to thoroughly test their endurance abilities, in which case, motivation is an important condition for the reliability of testing them [33]. Thus, the hypothesis about the negative impact of the first months of the pandemic on the examined characteristics was only confirmed by a significant increase in the waist circumferences of women and men. It is possible, however, that changes in somatic features are a kind of barometer announcing their later occurrence also in the motor sphere, which we noticed in our earlier studies of morphological changes and the output power of professional cyclists [34]. Therefore, we believe that research that includes the entire time of the pandemic and another period after its finish will allow for the real verification of this thesis.

The results of dimorphic differences observation complement the characteristics of temporal changes in somatic and motor traits of future physiotherapists. On their basis, it is difficult to indicate a clear tendency in the changes directions, which are observed in ontogenetic development [35]. In the case of body height, despite its slight changes in both sexes, a slight increase in the value of the dimorphism index results from a slightly higher body height of the examined women in the following years, and in the case of right-hand coordination and endurance measured by the time of 2 km walk—from a more visible deterioration of their results than in male groups. The identified picture of the dimorphic differences may be influenced by the stable development period of the studied adolescents, including both their somatic and motor features, as well as a slight influence of environmental conditions on the morphological and motor development of medical university students [27].

The results of the presented research and their analysis certainly do not exhaust the discussed problem and might most likely contain an error resulting from the limitations related to the observation of a relatively small number of characteristics of young people from only one university and carrying out two different years of study. Therefore, they cannot be fully representative of the Polish population of young people studying physiotherapy. However, they argue the need to continue research, including the assessment of the impact of the pandemic on the physical fitness of academic youth. Despite their relative value, they allowed for the formulation of the following conclusion below.

## 5. Conclusions

The long-term studies have shown the continued advantage of female participation in physiotherapy studies and no significant changes in height and weight of people of both sexes, as well as less favorable values than those found in previously studying adolescents, which proves the disappearance of the secular trend of these characteristics. There was also a time-dependent increase in waist circumference and a deterioration of the Cooper test results for women, which seems to be an unfavorable phenomenon and probably results from the lack of efficient identification for studies in this field, which was used in the previous decade. Therefore, it seems possible that some people studying physical therapy will have problems with the successful performance of therapeutic standards, which require considerable physical effort, without affecting their health.

## Figures and Tables

**Table 1:** Demographic characteristics in/of participants.

Years of Study	*n* (%)			Age (Mean ± SD)		
	All	Women	Men	All	Women	Men
2011	80	**52 (65.0)**	**28 (35.0)**	20.36 ± 0.83	20.23 ± 0.50	20.61 ± 1.20
2012	96	**66 (68.7)**	**30 (31.3)**	20.33 ± 0.80	20.29 ± 0.76	20.43 ± 0.90
2013	98	**67 (68.4)**	**31 (31.6)**	20.40 ± 1.00	**20.25 ± 0.77**	**20.71 ± 1.35**
2015	113	**83 (73.4)**	**30 (26.6)**	21.30 ± 0.72	21.3 ± 0.77	21.23 ± 0.57
2016	114	**88 (77.2)**	**26 (22.8)**	21.25 ± 0.63	21.25 ± 0.65	21.27 ± 0.60
2017	101	**75 (74,2)**	**26 (25,8)**	21.19 ± 0.50	21.19 ± 0.54	21.19 ± 0.40
2018	99	**76 (76,8)**	**23 (23.2)**	21.24 ± 0.73	21.22 ± 0.66	21.30 ± 0.93
2019	83	**57 (68.7)**	**26 (31.3)**	21.37 ± 0.85	21.30 ± 0.75	21.54 ± 1.03
2020	86	**60 (69.8)**	**26 (30.2)**	21.34 ± 0.82	**21.15 ± 0.61**	**21.77 ± 1.07**
all	870	**624 (71.7)**	**246 (28.3)**	20.99 ± 0.88	**20.95 ± 0.81**	**21.09 ± 1.02**
2001–2010	755	538 (71.3)	217 (28.7)	21.35 ± 1,33	21.32 ± 1,11	21.41 ± 1,75
D	115	−86 (−0.4)	–29 (0.4)	−0.36	−0.37	0.32
Z		k = 0.1308	k = 0.2095	6.3513	6.5778 *	2.3627 *

Bold type means comparisons of features of women and men for which statistical significance was found; * statistically significant difference *p* < 0.05 when comparing the mean values for 2011–2020 and 2001–2011; D—difference between arithmetic means; Z—the result of an independent test sample which is an asymptotic equivalent of the Student’s *t*-test; k—test result of the difference between uncorrelated proportions; *n* (%)—numerical and percentage characteristics of the size of student groups surveyed; mean—average age of student groups surveyed; SD—standard deviation.

**Table 2:** Statistical characteristics (mean ± SD) of body mass, height and BMI in participants.

Years of Study	Body Mass (kg)		Body Height (cm)		BMI (kg/m^2^)	
	Women	Men	Women	Men	Women	Men
2011	**60.36 ± 10.04**	**76.64 ± 8.44**	**166.62 ± 4.98**	**177.89 ± 6.84**	**21.74 ± 3.58**	**24.24 ± 2.51**
2012	**60.49 ± 8.13**	**79.05 ± 15.97**	**166.50 ± 5.87**	**180.45 ± 5.92**	**21.84 ± 2.93**	**24.25 ± 4.62**
2013	**60.87 ± 9.23**	**76.76 ± 11.47**	**167.97 ± 5.75**	**181.45 ± 6.74**	**21.54 ± 2.85**	**23.28 ± 2.99**
2015	**61.11 ± 8.00**	**78.67 ± 11.50**	**168.01 ± 6.16**	**179.07 ± 6.63**	**21.64 ± 2.50**	**24.50 ± 3.10**
2016	**61.14 ± 9.78**	**82.44 ± 15.66**	**165.86 ± 5.52**	**180.85 ± 7.94**	**22.23 ± 3.51**	**25.17 ± 4.34**
2017	**63.99 ± 11.94**	**81.88 ± 10.86**	**166.18 ± 5.81**	**178.79 ± 5.66**	**23.11 ± 3.70**	**25.62 ± 3.25**
2018	**61.66 ± 12.03**	**79.72 ± 10.48**	**167.16 ± 5.75**	**179.71 ± 6.70**	**22.09 ± 4.31**	**24.62 ± 2.29**
2019	**61.92 ± 8.65**	**79.74 ± 12.12**	**166.91 ± 5.75**	**178.63 ± 4.56**	**22.18 ± 2.59**	**24.97 ± 3.57**
2020	**61.09 ± 10.07**	**84.94 ± 12.86**	**168.15 ± 5.76**	**183.23 ± 5.38**	**21.58 ± 3.15**	**25.30 ± 3.78**
all	**61.45 ± 9.89**	**79.86 ± 12.45**	**167.02 ± 5.76**	**180.02 ± 6.43**	**22.02 ± 3.31**	**24.62 ± 3.49**
2001–2010	59.85 ± 8.76	76.79 ± 10.07	166.63 ± 6.08	179.95 ± 6.54	21.53 ± 2.78	23.69 ± 2.62
D	1.16	3.07	0.39	0.07	0.49	0.93
Z	3.0526 *	2.9311 *	1.1175	1.1158	2.9192 *	3.2654 *

Bold type means comparisons of the features of women and men for which statistical significance was found; * statistically significant difference *p* < 0.05 when comparing the means for 2011–2020 and 2001–2011; D—difference between arithmetic means; Z—the result of an independent test sample which is an asymptotic equivalent of the Student’s *t*-test; body mass—arithmetic mean and standard deviation of body mass of examined student groups; body height—arithmetic mean and standard deviation of body height of examined student groups; BMI—arithmetic mean and standard deviation of the body mass index of the examined student groups.

**Table 3:** Statistical characteristics (mean ± SD) of WHR index and waist and hip circumference.

Years of Study	Waist Circumference (cm)		Hip Circumference (cm)		WHR Index	
	Women	Men	Women	Men	Women	Men
2011	**67.94 ± 8.59**	**78.79 ± 6.0**	93.52 ± 7.15	95.82 ± 5.22	**0.72 ± 0.05**	**0.82 ± 0.03**
2012	**65.97 ± 5.49**	**82.93 ± 6.20**	**90.32 ± 5.87**	**95.40 ± 5.19**	**0.73 ± 0.06**	**0.87 ± 0.04**
2013	**65.55 ± 5.62**	**78.32 ± 7.40**	**91.29 ± 4.78**	**95.00 ± 5.00**	**0.71 ±** **0.05**	**0.82 ±** **0.05**
2015	**66.77 ± 5.45**	**79.0 ± 8.69**	**93.54 ± 5.69**	**97.13 ± 6.61**	**0.71 ± 0.03**	**0.81 ±** **0.05**
2016	**67.56 ± 7.82**	**82.5 ± 10.75**	**93.65 ± 7.32**	**98.65 ± 8.33**	**0.72 ±** **0.05**	**0.83 ±** **0.05**
2017	**68.85 ± 8.38**	**80.27 ± 8.32**	94.89 ± 8.66	97.92 ± 6.67	**0.72 ± 0.04**	**0.82 ±** **0.05**
2018	**68.03 ± 9.50**	**80.48 ± 8.57**	**93.79 ± 10.17**	**98.48 ± 6.61**	**0.73 ± 0.09**	**0.82 ± 0.06**
2019	**68.19 ± 7.73**	**80.92 ± 10.26**	95.26 ± 7.31	97.69 ± 5.66	**0.71 ± 0.04**	**0.83 ± 0.07**
2020	**75.18 ± 9.54**	**89.48 ± 9.82**	**92.39 ± 7.60**	**96.79 ± 7.44**	**0.81 ± 0.08**	**0.92 ± 0.06**
all	**68.10 ± 8.03**	**81.32 ± 8.97**	**93.21 ± 7.46**	**96.90 ± 6.36**	**0.73 ± 0.06**	**0.84 ± 0.06**
2001–2010	66.84 ± 6.88	78.19 ± 7.56	93.01 ± 6.39	95.25 ± 5.68	0,72 ± 0.06	0.82 ± 0.05
D	1.26	3.13	0.2	1.65	0.01	0.02
Z	2.8805 *	4.0726 *	0.4922	2.9483 *	2.8491 *	3.5746 *

Bold type means comparisons of the features of women and men for which statistical significance was found; * statistically significant difference *p* <0.05 when comparing the means for 2011–2020 and 2001–2011; D—difference between arithmetic means; Z—the result of an independent test sample which is an asymptotic equivalent of the Student’s *t*-test; waist circumference—arithmetic mean and standard deviation of waist circumferences of the examined student groups; hip circumference—arithmetic mean and standard deviation of hip circumferences of examined student groups; WHR index—arithmetic mean and standard deviation of waist–hip ratio of examined student groups.

**Table 4:** Statistical characteristics (mean ± SD) of fitness tests outcomes in research participants.

Years of Study	Cooper Test (m)		2 km March (s)		Turn Right		Turn Left	
	Women	Men	Women	Men	Women	Men	Women	Men
2011	**2148.86 ± 225.38**	**2488.64 ± 353.53**	**965.73 ± 56.09**	**915.75 ± 87.08**	351.60 ± 30.99	357.14 ± 45.35	355.15 ± 31.36	369.46 ± 48.86
2012	**2211.09 ± 366.38**	2641.07 ± 373.10	**965.18 ± 65.93**	**892.76 ± 83.66**	351.51 ± 36.06	358.93 ± 29.82	345.54 ± 38.78	364.67 ± 71.57
2013	**1987.10 ± 275.71**	**2387.10 ± 368.32**	**1029.09 ± 73.89**	**941.55 ± 85.80**	364.98 ± 59.26	387.19 ± 102.49	**360.57 ± 40.56**	**392.87 ± 77.00**
2015	**2077.02 ± 235.61**	**2471.26 ± 365.53**	**973.70 ± 61.20**	**926.33 ± 70.09**	339.99 ± 52.00	362.07 ± 53.82	**343.32 ± 41.15**	**373.03 ± 54.47**
2016	**2183.95 ± 260.23**	**2381.11 ± 299.84**	962.15 ± 75.52	956.04 ± 90.84	340.12 ± 41.84	350.42 ± 29.16	337.34 ± 39.71	346.00 ± 32.98
2017	**1927.61 ±** **260.94**	**2376.23 ± 340.29**	**1025.44 ± 77.53**	**927.46 ± 69.47**	338.21 ± 53.43	343.85 ± 40.97	338.44 ± 51.65	343.77 ± 39.90
2018	**1999.54 ± 261.02**	**2387.61 ± 338.45**	**956.32 ± 54.44**	**883.65 ± 51.57**	345.28 ± 55.69	363.78 ± 35.66	342.51 ± 59.42	356.04 ± 36.89
2019	**1991.77 ± 214.29**	**2399.42 ± 370.01**	**959.26 ± 55.40**	**897.88 ± 71.50**	343.75 ± 35.69	342.96 ± 43.77	346.35 ± 44.26	338.31 ± 54.20
2020	**2102.78 ± 264.30**	**2575.88 ± 356.12**	-	-	-	-	-	-
all	**2069.91 ± 280.94**	**2459.41 ± 359.68**	979.82 ± 71.59	918.37 ± 79.97	346.25 ± 47.93	358.94 ± 54.88	345.34 ± 44.90	361.70 ± 57.05
			1615.99 ± 119.25	1512.19 ± 133.55				
2001–2010	2046.9 ± 295.8	2519.1 ± 380.0	1602.4 ± 110.8	1502.4 ± 134.2	351.8 ± 44.2	365.2 ± 56.4	353.5 ± 42.8	367.4 ± 52.5
D	23.01	−59.69	13.59	9.79	−5.55	−6.26	−8.16	−5.70
Z	0.9282	1.7291	1.9607	0.7643	1.9993	1.1956	1.1345	1.0869

Bold type means comparisons of features of women and men for which statistical significance was found; D—difference between arithmetic means; Z—the result of an independent test sample which is an asymptotic equivalent of the Student’s *t*-test.

**Table 5:** The results of the analysis of temporal changes in the examined somatic traits in the decade 2011–2020.

Gender	Year_ Coef	Year_Coef *p*-Value	Year_ Std Err	Year_ 95% Cl	Const_ Coef	Const *p*-Value	Const Std Err	Const 95% Cl
**W—women body mass (R-squared = 0.002, F = 1.543, *p* (F) = 0.215)** **M—men body mass (R-squared = 0.024, F = 6.042, *p* (F) = 0.0147)**								
**W**	**0.2038**	**0.215**	**0.164**	**−0.118 0.526**	**−349.1958**	**0.291**	**330.558**	**−998.341 299.949**
**M**	**0.7498**	**0.015**	**0.305**	**0.149 1.351**	**−1430.9290**	**0.021**	**614.604**	**−2641.536 −220.322**
**W—women body height (R-squared = 0.000, F = 0.2903, *p* (F) = 0.590)** **M—men body height (R-squared = 0.006, F = 1.510, *p* (F) = 0.220)**								
**W**	**0.0515**	**0.590**	**0.096**	**−0.136 0.239**	**63.1532**	**0.743**	**192.769**	**−315.403 441.709**
**M**	**0.1952**	**0.220**	**0.159**	**−0.118 0.508**	**−213.3738**	**0.506**	**320.184**	**−844.051 417.304**
**W—women BMI (R-squared = 0.002, F = 1.036, *p* (F) = 0.309)** **M—men BMI (R-squared = 0.017, F = 4.202, *p* (F) = 0.0415)**								
**W**	**0.0560**	**0.309**	**0.055**	**−0.052 0.164**	**−90.7958**	**0.413**	**110.811**	**−308.405 126.813**
**M**	**0.1757**	**0.041**	**0.086**	**0.007 0.344**	**−329.3116**	**−0.058**	**172.668**	**−669.422 10.799**
**W—women waist circumference (R-squared = 0.049, F = 31.86, *p* (F) = 2.52 × 10^−8^)** **M—men waist circumference (R-squared = 0.041, F = 10.36, *p* (F) = 0.00146)**								
**W**	**0.7348**	**0.000**	**0.130**	**0.479 0.990**	**−1412.4769**	**0.000**	**262.315**	**−1927.608 −897.346**
**M**	**0.7015**	**0.001**	**0.218**	**0.272 1.131**	**−1332.1216**	**0.003**	**439.118**	**−2197.066 −467.177**
**W—women hip circumference (R-squared = 0.010, F = 6.125, *p* (F) = 0.0136)** **M—men hip circumference (R-squared = 0.016, F = 4.072, *p* (F) = 0.0447)**								
**W**	**0.3054**	**0.014**	**0.123**	**0.063 0.548**	**−522.2052**	**0.036**	**248.675**	**−1010.550 −33.861**
**M**	**0.3158**	**0.045**	**0.156**	**0.008 0.624**	**−539.3839**	**0.088**	**315.30**	**−1160.445 81.677**
**W—women WHR index (R-squared = 0.046, F = 29.97, *p* (F) = 6.37 × 10^−8^)** **M—men WHR index (R-squared = 0.035, F = 8.890, *p* (F) = 0.00316)**								
**W**	**0.0056**	**0.000**	**0.001**	**0.004 0.008**	**−10.5783**	**0.000**	**2.066**	**−14.635** **−6.522**
**M**	**0.0044**	**0.003**	**0.001**	**0.002 0.007**	**−8.0772**	**0.007**	**2.990**	**−13.967** **−2.187**

Year_coef: coefficient for the variable year; Year_coef *p*-value: *p*-value of the year coefficient; Year_std err: standard error for the year; Year_95% Cl: 95% confidence interval for the year factor; const_coef: the value of the model constant; const *p*-value: *p*-value of the model constant; const std err: standard error; Const 95% Cl 95% confidence interval for the constant value.

**Table 6:** The numerical characteristics of the temporal changes in the waist circumference of women and men in the decade 2011–2020.

W—Waist Circumference (R-squared = 0.008, F = 4.453, *p* (F) = 0.0353) M—Waist Circumference (R-squared = 0.001, F = 0.001, *p* (F) = 0.574)								
Gender	Year_Coef	Year_Coef *p*-Value	Year_ Std Err	Year_ 95% Cl	Const_ Coef	Const *p*-Value	Const Std Err	Const 95% Cl
**W**	**0.3099**	**0.035**	0.147	0.021 0.598	−557.0361	0.060	295.903	−1138.248 24.176
M	0.1405	0.574	0.250	−0.352 0.633	−202.6205	0.688	503.187	−1194.354 789.113

Year_coef: coefficient for the variable year; Year_coef *p*-value: *p*-value of the year coefficient; Year_std err: standard error for the year; Year_95% Cl: 95% confidence interval for the year factor; const_coef: the value of the model constant; const *p*-value: *p*-value of the model constant; const std err standard error; Const 95% Cl: 95% confidence interval for the constant value.

**Table 7:** The results of the analysis of temporal changes in the examined motor traits in the decade 2011–2020.

Gender	Year_ Coef	Year_ Coef *p*-Value	Year_ Std Err	Year_ 95% Cl	Const_ Coef	Const *p*-Value	Const Std Err	Const 95% Cl
**W—women Cooper test (R-squared = 0.021, F = 13.12, *p* (F) = 0.000316)** M—men Cooper test (R-squared = 0.004, F = 0.8653, *p* (F) = 0.353)								
**W**	**−16.7312**	**0.000**	4.619	−25.802 −7.660	3.578 × 10^4^	0.000	9307.797	1.75 × 10^4^ 5.41 × 10^4^
M	−8.2807	0.353	8.902	−25.815 9.254	1.914 × 10^4^	0.287	04 1.79 × 10^4^	−1.62 × 10^4^ 5.45 × 10^4^
W—women 2 km (s) (R-squared = 0.002, F = 1.169, *p* (F) = 0.280) M—men 2 km march calculated per seconds (R-squared = 0.003, F = 0.5729, *p* (F) = 0.450)								
W	−1.5239	0.280	1.409	−4.292 1.245	4049.8686	0.154	2839.455	−1527.371 9627.108
M	−1.8025	0.450	2.381	−6.496 2.891	4549.1610	0.344	4797.026	−4905.324 1.4 × 10^4^
**W—women spinning coordination—turn right (R-squared = 0.008, F = 4.812, *p* (F) = 0.0287)** M—men spinning coordination—turn right (R-squared = 0.013, F = 2.772, *p* (F) = 0.0974)								
**W**	**−2.0632**	**0.029**	0.941	−3.911 −0.216	4502.8322	0.018	1894.767	781.142 8224.523
M	−2.7076	0.097	1.626	−5.913 0.498	5812.9083	0.077	3275.905	−643.592 1.23 × 10^4^
**W—women spinning coordination—turn left (R-squared = 0.008, F = 4.327, *p* (F) = 0.0380)** **M—men spinning coordination—turn left (R-squared = 0.044, F = 10.06, *p* (F) = 0.00173)**								
**W**	**−1.8340**	**0.038**	0.882	−3.566 −0.102	4040.0193	0.023	1776.068	551.477 7528.562
**M**	**−5.2757**	**0.002**	1.663	−8.554 −1.997	1.099 × 10^4^	0.001	3350.732	4384.810 1.76 × 10^4^

Year_coef: coefficient for the variable year; Year_coef *p*-value: *p*-value of the year coefficient. Year_std err: standard error for the year; Year_95% Cl: 95% confidence interval for the year factor; const_coef: the value of the model constant; const *p*-value: *p*-value of the model constant; const std err: standard error; Const 95% Cl 95%: confidence interval for the constant value.

**Table 8:** Calculated Mollison indicators assessing dimorphic differences in motor and anthropometric characteristics.

Years of Study	Body Height	Body Mass	BMI	Waist Circum	Hip Circum	WHR Index	Cooper Test	2 km March	Turn Right	Turn Left
2011	−1.65	−1.93	−0.99	−1.81	−0.44	−3.33	−0.96	0.53	−0.12	−0.29
2012	−2.36	−1.16	−0.52	−2.73	−0.98	−3.50	−1.15	0.86	−0.25	−0.27
2013	−2.00	−1.38	−0.58	−1.72	−0.74	−2.20	−1.09	1.02	−0.22	−0.42
2015	−1.67	−1.53	−0.92	−1.41	−0.54	−2.00	−1.08	0.67	−0.41	−0.54
2016	−1.89	−1.36	−0.68	−1.39	−0.60	−2.20	−0.66	0.07	−0.35	−0.26
2017	−2.23	−1.64	−0.77	−1.37	−0.45	−2.00	−1.32	1.41	−0.14	−0.13
2018	−1.87	−1.72	−1.10	−1.45	−0.71	−1.50	−1.15	1.41	−0.52	−0.37
2019	−2.57	−1.47	−0.78	−1.24	−0.43	−1.71	−1.10	0.86	0.02	0.15
2020	−2.80	−1.91	−0.98	−1.46	−0.59	−1.83	−1.33	-	-	-

## Data Availability

Data supporting the findings of this study are available from the corresponding author upon request.
